# Sulfasalazine induced immune thrombocytopenia in a patient with rheumatoid arthritis

**DOI:** 10.1007/s10067-016-3420-9

**Published:** 2016-10-24

**Authors:** Nehal Narayan, Shirley Rigby, Francesco Carlucci

**Affiliations:** 1 Nuffield Orthopaedic Centre, Headington, Oxford, UK; 2 Warwick Hospital, Warwick, UK

**Keywords:** Rheumatoid arthritis (RA), Rheumatic diseases, Thrombocytopenia, Autoantibodies, Tissues or models, Hematologic diseases, Blood platelets disorders

## Abstract

Sulfasalazine has long been used for the treatment of rheumatoid arthritis and is often chosen as a first-line treatment. Here, we report a case of sulfasalazine-induced autoimmune thrombocytopenia and review the mechanisms behind drug-induced immune thrombocytopenia (DITP) and the approach to its diagnosis and management.

Sulfasalazine (SSZ) has been used for the treatment of rheumatoid arthritis (RA) for decades. Given its safety profile (especially lack of teratogenicity), ease of administration and cost, it is often used as first line treatment. Myelosuppression is a recognized side effect of sulfasalazine, as is the development of drug induced-Systemic Lupus Erythematosus (SLE) or SLE-like disease [1], which may also result in blood dyscrasias. Here, we report a case of sulfasalazine induced autoimmune thrombocytopenia, which rapidly settled on stopping the drug, highlighting the importance of considering this rare complication of sulfasalazine therapy. We also review the mechanisms behind drug induced immune thrombocytopenia (DITP) and the approach to its diagnosis and management.

Our patient, a 67 year old Caucasian man, presented with symmetrical synovitis of knees, shoulders, and small joints of the hands. A diagnosis of seronegative RA was made. ANA was negative at time of diagnosis. He was commenced on sulfasalazine, with remission of arthritis 4 months later. His other medication consisted of diclofenac 50 mg, which he took up to twice a day, as needed. Two years after diagnosis, at a routine follow up, it was noted that his platelet count had been steadily declining for 3 months, from an average count of 230 × 10^9^/L to 90 × 10^9/^L. The patient himself was well, without rash, symptoms of bleeding, or history of recent illness and his RA remained in remission. Medication was unchanged. Examination was unremarkable; the patient was afebrile, with no evidence of purpura, or bleeding, and no lymphadenopathy or hepatosplenomegaly.

Repeat platelet count was 87 × 10^9/^L. Rest of the full blood count, peripheral blood film, and INR (international normalized ratio) were unremarkable. Sulfasalazine was stopped, and the patient continued to take diclofenac as needed.

Immunology testing demonstrated negative ANA, but direct anti-platelet IgM and IgG were positive, consistent with autoimmune thrombocytopenia.

Two weeks after SSZ was stopped, the platelet count had increased to 150 × 10^9/^L. Three months later, platelet count was steady at 200 × 10^9/^L, and Anti-platelet IgM was negative. The patient’s arthritis remained in remission, and no further DMARDs were initiated.

Only one case report exists that describes an association of sulfasalazine with an autoimmune thrombocytopenia, although that case was also associated with positivity to lupus anticoagulant. [2] Although NSAIDs are a reported cause of thrombocytopenia, in our case, this is extremely unlikely to be the causative drug, since diclofenac was continued while sulfasalazine was ceased, and platelet count still normalized.

Thrombocytopenia is defined as a platelet count of below 150 × 10^9^/L. Prediction of bleeding risk by platelet count is unreliable [3], but generally, platelet counts above 50 × 10^9^/L usually do not lead to bleeding, unless platelet dysfunction is also present. At platelet counts of less than 30 × 10^9^/L, spontaneous bleeding and purpura may occur, with clinically significant bleeding occurring when count is under 10 × 10^9^/L [4]. Thrombosis may also be associated with a low platelet count, particularly in heparin-induced thrombocytopenia.

Drug induced immune thrombocytopenia (DITP) is a rare cause of immune thrombocytopenia, with an estimated incidence of ten cases per million per year [5], and is most common in critically unwell, hospitalized patients. DITP has been reported to occur via at least six different mechanisms [6, 7] (see Table 1).

**Table 1:** Mechanisms of DITP and examples of precipitating medications [6, 7]

Mechanism	Example(s)
Autoantibody induction	
Drug induces direct anti-platelet antibodies that adhere to their target in the absence of soluble drug in serum	Gold salts, penicillamine
Drug dependent antibody induction	
Drug induces antibodies that bind to platelet membrane proteins only in the presence of soluble drug in the serum. Presence of circulating drug-dependent antibodies is highly indicative of the diagnosis	NSAIDs, quinine, sulfonamides, carbamazepine
Hapten-dependent antibody induction	
Drug links covalently to platelet membrane proteins and induces a drug specific immune response	Penicillin, cephalosporins
Immune Complex formation	
Drugs bind to Platelet Factor 4, producing a complex on the surface of platelets, to which the patient generates antibodies, resulting in both platelet activation and destruction	Heparin
Fiban-induced thrombocytopenia	
Drug reacts with platelet membrane glycoprotein IIb/IIIa and induces a conformational change, recognized by patient’s existing antibodies	Tirofiban
Drug-specific antibody induction	
Naturally occurring or induced antibody is specific for a murine component of abiciximab, a chimeric antibody therapy to GPIIIa, present on platelets	Abciximab

Testing for specific antibodies for DITP, such drug dependent antibodies can be complex and time consuming, and is available only in a few laboratories. Therefore, a set of clinical criteria have been proposed as a more practical way of establishing the presence of drug induced ITP based on systematic review of existing literature [8] (see Table 2).

**Table 2:** Proposed criterion for the diagnosis of DITP. Definite diagnosis: criteria 1, 2, 3 and 4 are met, diagnosis probable: criteria 1, 2 and 3 are met; diagnosis possible: criteria 1 met; diagnosis unlikely: criterion 1 not met [8]

Criterion	Description
1	Therapy with suspected drug preceded thrombocytopenia and recovery from thrombocytopenia was complete and sustained after discontinuation of the suspected drug
2	The suspected drug was the only drug used before the onset of thrombocytopenia or other drugs were continued or re-introduced after discontinuation with the suspected drug, with a sustained normal platelet count
3	Other causes of thrombocytopenia have been excluded
4	Response to the suspected drug resulted in recurrence of the thrombocytopenia

The management of a patient with suspected thrombocytopenia is aimed at establishing the cause, and assessing and treating those with, or at risk of, clinically significant bleeding. History taking includes questioning for evidence of bleeding, constitutional symptoms such as fevers, sweats, weight loss, which may indicate an underlying neoplastic or infective cause for low platelets, and a drug history to look for pre-disposing medications. Examination should look for purpura and other signs of bleeding, as well as hepatosplenomegaly and lymphadenopathy to look for evidence of myelodysplasia.

Initial investigations should include a full blood count, to rule out leukopenia and anaemia. A peripheral blood film is essential, to assess for falsely low platelet count secondary to platelet clumping. Clotting tests such as INR are useful, to assess bleeding risk further, and viral screen for HIV and Hepatitis C should be considered [4].

Management of DITP is to stop the precipitating drug. For those with a platelet count of above 10 × 10^9^/L without bleeding, or other risk factors for bleeding, monitoring of the platelet count may be all that is required, with rapid recovery of platelet count within days to weeks as drug is cleared. For those with platelets of less than 10 × 10^9/^L, and/or with bleeding, or risk factors for bleeding, such as sepsis, or other blood abnormalities, platelet transfusion is considered [9]. Use of corticosteroids, intravenous immunoglobulins, and plasma exchange, has also been described, but the benefit of these is uncertain [6].
